# Dual Modulation of Senescence and Immune Checkpoints by Metformin and Dapagliflozin Attenuates Liver Fibrosis in a Thioacetamide-Induced Rat Model

**DOI:** 10.34172/apb.025.46231

**Published:** 2025-12-22

**Authors:** Asmaa Ramadan, Ahmed A. Shaaban, Mohammad E. Rabeh, Ahmad M. Rabi, Eslam E. Abd El-Fattah, Amal K. Seleem, Ibrahim Osman, Ayman Salama, Noha M. Gamil, Ahmed S.G. Srag El-Din

**Affiliations:** ^1^Department of Biochemistry, Faculty of Pharmacy, Delta University for Science and Technology, Gamasa, Egypt; ^2^Pharmacology and Toxicology Department, Faculty of Pharmacy, Mansoura University, Mansoura, Egypt; ^3^Faculty of Pharmacy, Jerash University, Jerash, Jordan; ^4^Beckman Research Institute, City of Hope, Duarte, USA; ^5^Medical Biochemistry and Molecular Biology, Faculty of Medicine, Mansoura University, Mansoura, Egypt; ^6^Faculty of Pharmacy, Delta University for Science and Technology, Gamasa, Egypt; ^7^Department of Pharmaceutics, Faculty of Pharmacy, University of Tabuk, Tabuk, Saudi Arabia; ^8^Department of Pharmacology and Toxicology, Faculty of Pharmaceutical Sciences and Drug Manufacturing, Misr University for Science and Technology, 6th of October City, Egypt; ^9^Department of Pharmaceutics, Faculty of Pharmacy, Delta University for Science and Technology, Gamasa, Egypt; ^10^Department of Pharmaceutics, College of Pharmacy, Almaaqal University, 61014 Basrah, Iraq

**Keywords:** Liver fibrosis, Metformin, Dapagliflozin, Senescence, PD-L1

## Abstract

**Introduction:**

Liver fibrosis, characterized by excessive extracellular matrix accumulation, reflects a maladaptive repair response to persistent hepatic injury. Recent studies implicate cellular senescence and immune checkpoint dysregulation as pivotal drivers of fibrogenesis, yet these pathways remain underutilized in therapeutic development. This study evaluates the anti-fibrotic efficacy of metformin and dapagliflozin, two metabolic modulators, in a thioacetamide (TAA)-induced rat model, emphasizing their impact on senescence and immune regulation.

**Methods:**

Male albino rats (N=6/group) were assigned to five groups: control, TAA (200 mg/kg, i.p., thrice weekly for 4 weeks), TAA+metformin (300 mg/kg/day), TAA+dapagliflozin (1 mg/kg/day), and TAA+combination therapy. Liver tissues underwent histopathological examination and SMAD-3 mRNA quantification via qRT-PCR. Protein levels of TGF-β1, PD-1, p16, NF-κB, α-SMA, fibronectin, collagen-I, and sirtuin-1 were assessed, alongside serum oxidative stress markers (MDA, SOD) and liver enzymes (ALT, AST).

**Results:**

All treatments significantly reduced fibrosis, collagen deposition and improved liver architecture. Mechanistically, both drugs suppressed fibrotic and senescence markers, downregulated PD-1, enhanced sirtuin-1, and mitigated oxidative stress. Notably, combination therapy yielded synergistic anti-fibrotic effects.

**Conclusions:**

These findings highlight a dual-pathway therapeutic strategy targeting senescence and immune imbalance, with relevance to metabolic liver disease.

## Introduction

 Liver fibrosis is a critical pathological process that underpins the progression to end-stage liver disease, posing a significant worldwide health concern. Epidemiological data reveal its rising burden, with chronic liver disorders altogether responsible for more than 1.9 million deaths globally annually.^1^ Fibrogenesis is a progressive and dynamic condition that emerges from diverse etiological factors, including metabolic dysfunction-associated steatotic liver disease (MASLD), chronic viral hepatitis, and alcohol-related liver injury. All of these converge on a final common pathway characterized by excessive extracellular matrix (ECM) deposition and architectural distortion of the hepatic parenchyma. The clinical implications of fibrosis are particularly relevant in the context of metabolic diseases, where obesity has been shown to significantly increase fibrosis prevalence to 14.7%, compared with only 2.1% among normal-weight people.^2^ Furthermore, advanced stages of liver fibrosis are frequently reported in patients with type 2 diabetes maintained in outpatient treatment, underlining the interdependent link between metabolic dysregulation and hepatic fibrogenesis.^3^

 Liver fibrosis is caused by two key biological processes: immune microenvironment dysregulation and cellular senescence. The liver balances immunological tolerance and inflammation during normal physiology.^4^ Chronic damage upsets the balance by activating TGF-β signaling, which activates hepatic stellate cells (HSCs) and causes myofibroblast differentiation. ^5,6^ Recent research has shown that immunological checkpoint molecules, including programmed cell death protein 1 (PD-1), have an additional role in inducing fibrogenesis by modulating fibroblast activity.^7^

 Parallel to immune dysregulation, cellular senescence has emerged as a key contributor to fibrotic progression. Senescent cells exhibit characteristic cell cycle arrest and secrete pro-fibrotic factors through the senescence-associated secretory phenotype (SASP).^8^ Notably, markers of hepatocyte senescence strongly correlate with hepatic fat accumulation, and targeted elimination of senescent cells has shown therapeutic potential in experimental models.^9^ These findings position senescence as a promising target for anti-fibrotic interventions.

 Recent pharmacological advances have identified unexpected anti-fibrotic properties in existing metabolic drugs. Metformin, a first-line treatment for type 2 diabetes, demonstrates potent anti-fibrotic effects through AMP-activated protein kinase (AMPK) activation and subsequent inhibition of nuclear factor kappa B (NF-κB) signaling, one of the main inducers of SASPs secretion.^10^ Similarly, sodium-glucose cotransporter-2 (SGLT2) inhibitors like dapagliflozin exhibit pleiotropic benefits beyond glycemic control, including antioxidant and anti-inflammatory properties that attenuate liver fibrosis.^11^ Intriguingly, both drug classes may modulate cellular senescence, with metformin reducing oxidative stress-related senescence,^12^ and dapagliflozin enhancing senescent cell clearance.^13^

 Recent studies have demonstrated that metformin and dapagliflozin exert pleiotropic effects that extend beyond their antidiabetic properties. Metformin activates AMP-activated protein kinase (AMPK), a key regulator of cellular energy homeostasis, which has been shown to inhibit hepatic stellate cell activation and reduce fibrogenesis. Additionally, metformin modulates SIRT1 signaling and suppresses TGF-β1-mediated profibrotic pathways.^14-17^ Dapagliflozin, a selective SGLT2 inhibitor, has been reported to attenuate oxidative stress, inflammation and may exert antifibrotic effects via modulation of AMPK and reduction of hepatic lipid accumulation.^18^ These mechanisms suggest a potential therapeutic role for both agents in mitigating liver fibrosis, particularly in toxin-induced models such as TAA.

 Nevertheless, significant knowledge gaps remain, particularly regarding the molecular interplay between cellular senescence and immune checkpoint regulation in the etiology of liver fibrosis and the potential synergistic efficacy of metformin and dapagliflozin therapy. The current investigation aimed to fill these gaps by comprehensively assessing the effects of these pharmacological treatments on senescence-associated markers and critical immune microenvironmental components in a well-validated mouse model of liver fibrosis. The findings reveal a dual-pathway mechanism by which these repurposed metabolic modulators exert anti-fibrotic effects, providing novel translational insights with potential therapeutic relevance for fibrotic liver diseases, especially in the presence of concomitant metabolic dysfunction.

## Materials and Methods

###  Drugs

 Metformin Hydrochloride (Cidophage® 1000 mg) was obtained from Chemical Industries Development Company (CID, Cairo, Egypt). Dapagliflozin (Forxiga, 10 mg) was obtained from AstraZeneca Pharmaceuticals, Inc. (Egypt). Thioacetamide (TAA) was obtained from Sigma-Aldrich Inc., Missouri, USA (Cat number 163678, CAS 62-55-5).

###  Experimental Design

 Thirty adult male Sprague Dawley rats (250 ± 50 g) were obtained from the National Research Center (NRC, Giza, Egypt) and acclimatized for one week before experimentation. All procedures were conducted in compliance with international ethical standards and approved by the Institutional Animal Care and Use Committee of Delta University for Science and Technology (Approval No. FPDu 15/2024), adhering to ARRIVE guidelines, EU Directive 2010/63/EU, U.K. Animals (Scientific Procedures) Act, 1986, and the NIH Guide for Care and Use of Laboratory Animals. Animals were housed in controlled environmental conditions (22 ± 2°C, 12-h light/dark cycle) with ad libitum access to standard chow and water. Following simple randomization, rats were divided into five experimental groups (n = 6 per group), which aligns with prior studies of the TAA-induced fibrosis model.^19,20^ Drug doses were selected based on previous pharmacological studies demonstrating efficacy in liver fibrosis models as follow:

 The control group (N) received vehicle therapy (0.5% CMC, p.o. daily), whereas the fibrosis model group (T) was given thioacetamide (TAA; 200 mg/kg, i.p., three times weekly).^21^ The metformin-treated group (M) received TAA in addition to metformin (300 mg/kg, p.o. daily),^22^ whereas the dapagliflozin-treated group (D) received TAA plus dapagliflozin (1 mg/kg, p.o. daily).^23^ The combination group (DM) received TAA, metformin, and dapagliflozin at the aforementioned doses. The treatment duration was 4 weeks for all groups. Blinding was implemented during data collection and analysis to minimize observer bias; investigators assessing outcomes were unaware of group assignments.

###  Collection of Blood Samples and Liver Tissues

 After the study’s completion, all rats were anesthetized using thiopental (100 mg/kg/i.p.),^24^ euthanized by cervical dislocation, and their livers were dissected. Following blood collection, serum was extracted and stored at -80°C for biochemical analysis. The fresh livers were washed with ice-cold saline and dried on a clean paper towel. The liver was divided into two sections: one was stored in 10% formalin saline for histological investigation and immunohistochemical analysis, whereas the other was promptly frozen in liquid nitrogen and stored at -80 °C for reverse transcription polymerase chain reaction gene expression, enzyme-linked immunosorbent assay (ELISA), western blot, and colorimetric experiments.

###  Histopathological Examination

 Liver tissues kept in formalin were fixed into paraffin blocks, sliced into 5-μm-thick sections, and stained with hematoxylin and eosin. The slides were inspected blindly under a light microscope by a pathologist, and the images were recorded using a digital image-capture system.

###  Biochemical Analysis

####  ELISA Assessment of TGFβ1, β-Catenin, 

 According to the manufacturer’s instructions, β-catenin and TGFβ1 levels in liver tissues were assessed using ELISA kits: β-catenin (MyBioSource, CA, USA; Cat number MBS7269476) and TGFβ1 (Finetest, China; Cat number ER1378).

####  Immunohistochemical Assessment of SIRT-1, Fibronectin, SMA-α, and Collagen Production

 The hepatic expressions of SIRT-1 (Abcam, Cambridge, UK; Cat number ab110304), fibronectin (ABclonal, USA; Cat number A12932), SMA-α (Servicebio, Wuhan, China; Cat number GB111364), and collagen (Masson trichrome stain; Sigma-Aldrich, St. Louis, MO, Cat number HT15-1KT) production in treated and untreated rat liver tissues were evaluated using immunohistochemistry. Sections of tissue with a thickness of 5 μm were deparaffinized, rehydrated, and then heated in citrate buffer to retrieve antigen. Hydrogen peroxide therapy inhibited endogenous peroxidases. To find SIRT-1, fibronectin, SMA-α, and collagen production, the sections were then incubated for a whole night at 4°C using a polyclonal rabbit primary antibody for each protein (1:100 dilution). Using DAB as the chromogen and a goat anti-rabbit secondary antibody coupled with horseradish peroxidase, binding was observed. Using ImageJ 1.54f software, digital images from ten randomly selected high-power fields per section were captured and blindly analyzed. Quantitative image analysis was performed to measure variations in SIRT-1, fibronectin, α-SMA, and collagen expression among the experimental groups, offering information on fibrosis and senescence signaling. Furthermore, Liver fibrosis was assessed using blinded histologic scoring of hematoxylin and eosin and Masson’s trichrome-stained sections. Fibrosis severity was graded according to established semi-quantitative scoring systems validated for TAA-induced liver injury models.^19,25^ Collagen deposition was further quantified using the Fibrosis Index, calculated as the percentage of collagen-positive area in Masson’s trichrome images through digital morphometric analysis, providing an objective and reproducible measure of collagen accumulation.

####  Western blot Assessment of NF-κB, PD-1, and P16 Levels

 Liver tissues were washed with PBS, homogenized, and lysed with RIPA buffer containing protease and phosphatase inhibitor cocktail. Protein concentrations were quantified using the Pierce^TM^ BCA Protein Assay Kit. An equal volume (30–50 μg) of protein lysates was loaded and separated using the TGX Stain-Free^TM^ FastCast^TM^ Acrylamide Kit (SDS-PAGE, Bio-Rad, USA) and then transferred to 0.2-μm nitrocellulose membranes in an X-cell II apparatus. After blocking membranes with 5% non-fat milk with TBST (1X TBS, 0.1% Tween 20) for 1 h, membranes were incubated overnight at 4°C with primary antibodies for NF-κB (Cell Signaling, USA; Cat number 3033), PD-1 (Elabscience, USA; Cat number E-AB-70227), and P16 (Santa Cruz Biotechnology, USA; Cat number sc-56330). The next day, membranes were washed three times (5 min each) with TBST and then incubated with appropriate horseradish peroxidase (HRP)-conjugated secondary antibodies for ⁓2 h at room temperature. Membranes were washed three times (5 min each) with TBST. The band intensity was quantified using a Java-based image processing program, Image J 1.52a and normalized against corresponding anti-β-actin.

####  Quantitative Real-Time Polymerase Chain Reaction (PCR) of SMAD-3 in Liver Tissue

 The NucleoSpin® kit was used to extract total RNA from liver tissue, which was then reverse-transcribed into cDNA and amplified using the SensiFAST^TM^ SYBR® Hi-ROX One-Step Kit (Bioline, Meridian Bioscience GmbH, Luckenwalde, Germany; Cat number BIO-73001). The 2^-ΔΔCt^ method was used to calculate the relative expression of each target gene mRNA, normalized to glyceraldehyde-3-phosphate dehydrogenase (GAPDH). The primer sequences for the targeted genes are listed in Table 1.

**Table 1:** Primers for the studied genes.

**Gene**	**Forward primer**	**Reverse primer**
SMAD-3	5’-GGCTTTGAGGCTGTCTACCA-3’	5’-GGTGCTGGTCACTGTCTGTC-3’
GAPDH	5’-ATGGTGAAGGTCGGTGTGAACG-3’	5’-TGGTGAAGACGCCAGTAGACTC-3’

####  Assessment of Oxidative Stress Markers (GSH and MDA)

 Reduced glutathione (GSH) and malondialdehyde (MDA) levels were quantified in liver tissue homogenates using commercial spectrophotometric kits (Bio-diagnostic, Giza, Egypt, Cat. nos. GR 25 11 for GSH and MD 25 29 for MDA, respectively). MDA was measured at 534 nm against a TEP standard curve, while GSH was measured at 412 nm against a GSH standard curve. All results were expressed as nmol/mg protein, following the manufacturer’s specified protocols.

####  Assessment of Liver Enzymes (ALT and AST)

 Alanine transaminase (ALT) and aspartate transaminase (AST) serum levels were assessed, and all procedures were performed as per Elitech Clinical Systems (Elitech Group, Paris, France) manufacturer’s instructions.

###  Statistical Analysis

 Statistical analysis was conducted using GraphPad Prism software version 6 (GraphPad Software Inc., La Jolla, CA, USA), and the data are presented as the mean ± standard deviation (SD). Normality of data distribution was assessed using the Shapiro–Wilk test. Differences between groups were analyzed by a one-way analysis of variance followed by Tukey’s post hoc for multiple comparisons test. P values < 0.05 were considered statistically significant.

## Results

###  Effect of Metformin and Dapagliflozin Treatment on Histopathological Characteristics (H & E)

**Figure 1:**
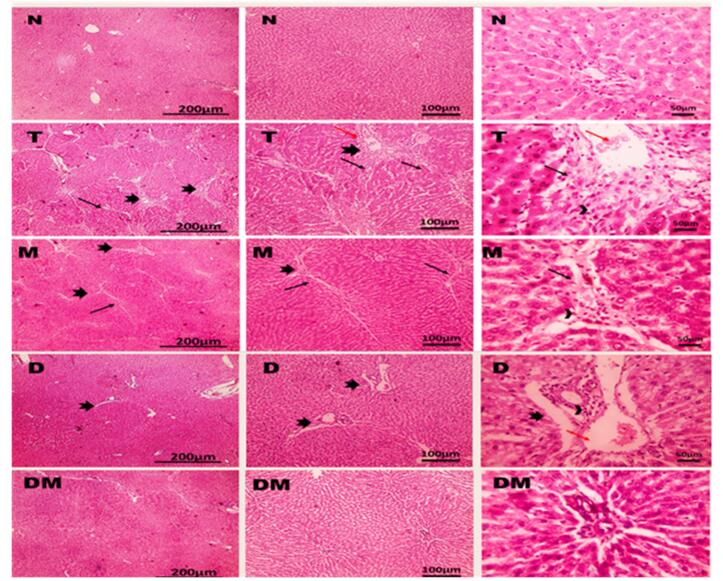
Microscopic pictures of HE-stained liver sections showing a normal arrangement of hepatocytes, normal central veins, portal areas, and sinusoids in the control group (N). Liver sections from the thioacetamide diseased group (T) showing portal fibrosis (thick black arrow), congestion (red arrow), and inflammation (arrowhead), sending thick anastomosing fibrous tissue extensions (thin black arrow) into hepatic parenchyma. Liver sections from metformin-treated group (M) showed mild portal fibrosis (thick black arrow) with mild portal inflammation (arrowhead), sending long, thin, non-anastomosing fibrous tissue extensions (thin black arrow) into hepatic parenchyma. Liver sections from dapagliflozin-treated group (D) showed mild portal fibrosis, mild portal inflammation (arrowhead), mild portal edema (thick black arrow), and congestion (red arrow). Liver sections from the combination-treated group DM showing repaired hepatic parenchyma with no signs of portal fibrosis. Magnifications of X: 40 bar 200, X: 100 bar 100, and X: 400 bar 50

 As depicted in Figure 1, microscopic pictures of H&E-stained liver sections show a normal arrangement of hepatocytes, normal central veins, portal areas, and sinusoids in the control group. Liver sections from the diseased group showing portal fibrosis (thick black arrow), congestion (red arrow), and inflammation (arrowhead), sending thick anastomosing fibrous tissue extensions (thin black arrow) into hepatic parenchyma. Liver sections from the treated group M show mild portal fibrosis (thick black arrow) with mild portal inflammation (arrowhead) sending long, thin, non-anastomosing fibrous tissue extensions (thin black arrow) into the hepatic parenchyma. Liver sections from the treated group D show mild portal fibrosis, mild portal inflammation (arrowhead), mild portal edema (thick black arrow), and congestion (red arrow). Liver sections from the combination-treated group DM show repaired hepatic parenchyma with no signs of portal fibrosis.

###  Effects of Metformin and Dapagliflozin Treatment on Collagen Synthesis and SMA-α

**Figure 2:**
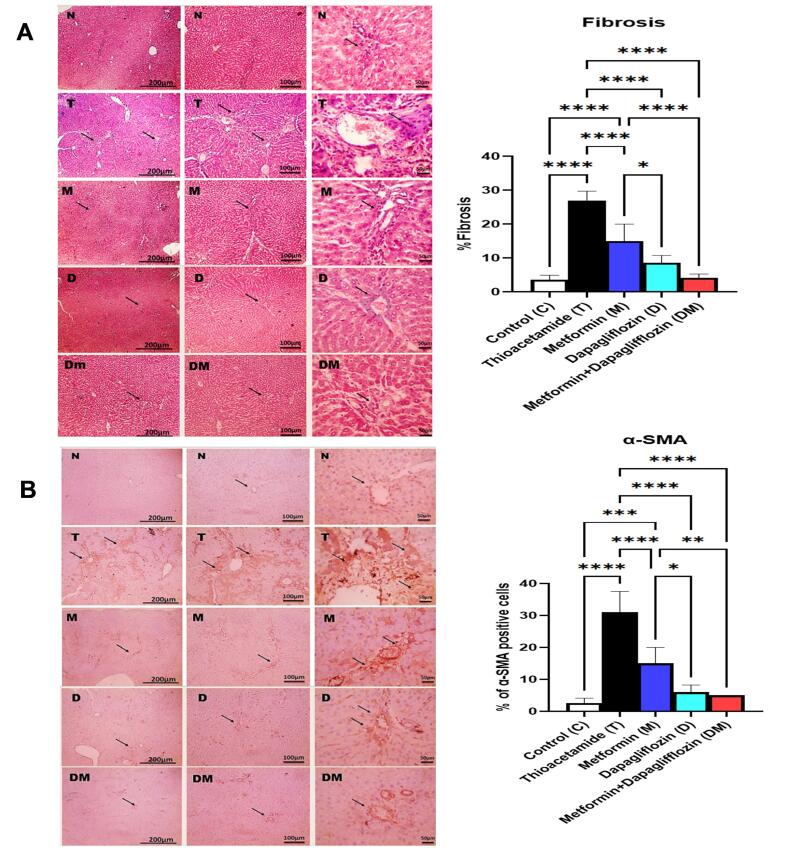
(A) Microscopic pictures of Masson Trichrome stained liver sections showing no signs of portal fibrosis (black arrow) in the control group (N). Liver sections from the diseased group showing prominent bluish portal fibrosis (black arrow). Liver sections from Metformin treated group (M) showing mild bluish portal fibrosis (black arrow). Liver sections from the dapagliflozin-treated group (D) showing milder bluish portal fibrosis (black arrow). Liver sections from the combination-treated group DM showing no signs of portal fibrosis (black arrow). (B) Microscopic pictures of immunostained liver sections against SMA-α showing mild positive brown expression around blood vessels (black arrow) in the control group. Liver sections from the thioacetamide diseased group (T) showing prominent positive brown expression in portal areas and surrounding hepatic parenchyma (black arrow). Liver sections from the metformin-treated group (M) showing decreased positive brown expression in portal areas and surrounding hepatic parenchyma (black arrow). Liver sections from the dapagliflozin-treated group (D) showing mild positive brown expression in portal areas and surrounding hepatic parenchyma (black arrow). Liver sections from the combination-treated group with DM showing mild positive brown expression in portal areas (black arrow). Data are presented as the mean ± SD (n = 6 per group). SMA-α: Alpha Smooth Muscle Actin. ****P < 0.0001, ***P < 0.001, **P < 0.01, *P < 0.05. Control (C): normal rats received the CMC vehicle orally daily for one month; Thioacetamide (T): Rats received thioacetamide (200 mg/kg) i.p. three times weekly for 4 weeks; Metformin (M): thioacetamide-induced liver fibrosis rats treated with oral metformin (300 mg/kg) daily for 4 weeks; Dapagliflozin (D): thioacetamide-induced liver fibrosis rats treated with oral dapagliflozin (1 mg/kg) daily for 4 weeks; The combination group (DM): thioacetamide-induced liver fibrosis rats treated with oral dapagliflozin (1 mg/kg) and oral metformin (300 mg/kg) daily for 4 weeks

Figure 2A shows that the fibrosis percentage was significantly increased in the liver tissue of rats of the thioacetamide group (7.5-fold) compared to the control group. At the same time, M, D, and DM reversed the thioacetamide effect (44%, 68.14%, and 84.4%), respectively. Besides, Figure 2B showed that the percentage of SMA-α positive cells significantly increased in the thioacetamide group (11.92-fold) versus the control group, which was reversed by M, D, and DM by 51.6%, 80.64%, and 83.8%, respectively.

###  Effects of Metformin and Dapagliflozin Treatment on Fibronectin

**Figure 3:**
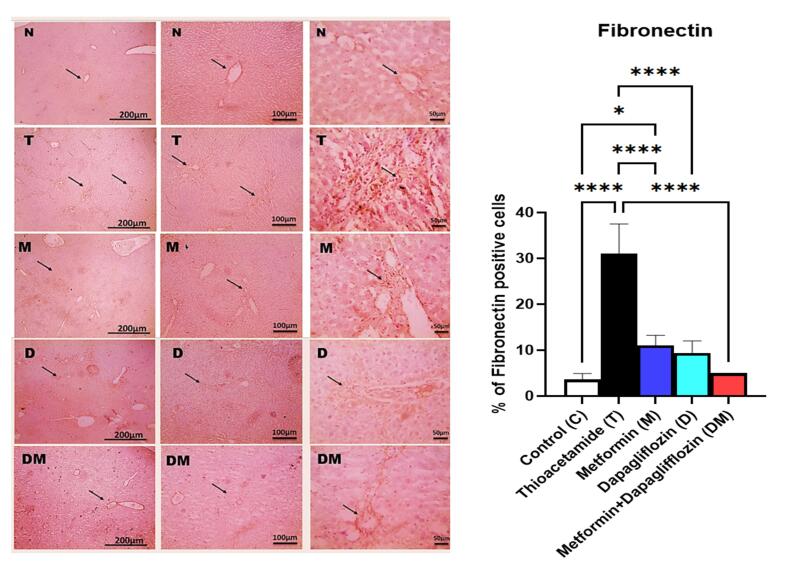
Microscopic pictures of immunostained liver sections against fibronectin showing mild positive brown expression around blood vessels (black arrow) in the control group (N). Liver sections from the thioacetamide-diseased group (T) showing prominent positive brown expression in portal areas (black arrow). Liver sections from the metformin-treated group (M) showing decreased positive brown expression in portal areas (black arrow). Liver sections from the dapagliflozin-treated group (D) showing mild positive brown expression in portal areas (black arrow). Liver sections from the combination-treated group DM showing mild positive brown expression in portal areas (black arrow). Magnifications of X: 40 bar 200, X: 100 bar 100, and X: 400 bar 50. Data are presented as the mean ± SD (n = 6 per group). ****P < 0.0001, ***P < 0.001, **P < 0.01, *P < 0.05. Control (C): normal rats received the CMC vehicle orally daily for one month; Thioacetamide (T): Rats received thioacetamide (200 mg/kg) i.p. three times weekly for 4 weeks; Metformin (M): thioacetamide-induced liver fibrosis rats treated with oral metformin (300 mg/kg) daily for 4 weeks; Dapagliflozin (D): thioacetamide-induced liver fibrosis rats treated with oral dapagliflozin (1 mg/kg) daily for 4 weeks; The combination group (DM): thioacetamide-induced liver fibrosis rats treated with oral dapagliflozin (1 mg/kg) and oral metformin (300 mg/kg) daily for 4 weeks

 The percentage of fibronectin-positive cells significantly increased in the thioacetamide group (8.6-fold) versus the control group, in which M, D, and DM decreased fibronectin levels by 64.5%, 69.67%, and 83.87%, respectively (Figure 3).

###  Effect of Metformin and Dapagliflozin Treatment on Senescence (SIRT-1 and P16)

**Figure 4:**
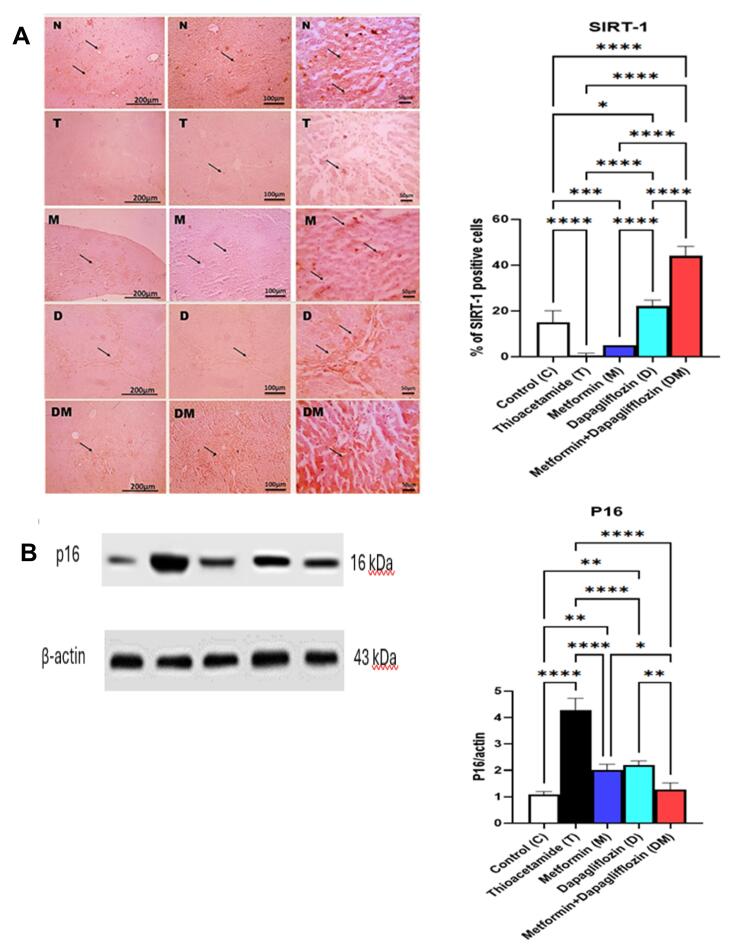
(A) Microscopic pictures of immunostained liver sections against Sirt-1 showing positive brown expression in hepatocytes (black arrow) around blood vessels in the control group (N). Liver sections from the diseased group show markedly decreased positive brown expression in hepatocytes (black arrow). Liver sections from the metformin-treated group (M) showing increased positive brown expression in hepatocytes (black arrow). Liver sections from the dapagliflozin-treated group (D) showing more increased positive brown expression in hepatocytes (black arrow). Liver sections from the combination-treated group DM showing markedly increased positive brown expression in hepatocytes (black arrow). Magnifications are X: 40 bar 200, X: 100 bar 100, and X: 400 bar 50. (B) Western blotting analysis of P16 in the studied groups. Data are presented as the mean ± SD (n = 6 per group). SIRT-1: Sirtuin-1. ****P < 0.0001, ***P < 0.001, **P < 0.01, *P < 0.05. Control (C): normal rats received the CMC vehicle orally daily for one month; Thioacetamide (T): Rats received thioacetamide (200 mg/kg) i.p. three times weekly for 4 weeks; Metformin (M): thioacetamide-induced liver fibrosis rats treated with oral metformin (300 mg/kg) daily for 4 weeks; Dapagliflozin (D): thioacetamide-induced liver fibrosis rats treated with oral dapagliflozin (1 mg/kg) daily for 4 weeks; the combination group (DM): thioacetamide-induced liver fibrosis rats treated with oral dapagliflozin (1 mg/kg) and oral metformin (300 mg/kg) daily for 4 weeks

Figure 4A shows that the Sirtuin-positive cells were significantly decreased in the liver tissue of rats of the thioacetamide group (96%) compared to the control group. At the same time, M, D, and DM increased their level by 8.33-fold, 36.66-fold, and 73.33-fold, respectively. Figure 4B showed that the P16 protein level was significantly increased in the thioacetamide group (3.91-fold) versus the control group, which was reversed by M, D, and DM by 52.9%, 48.56%, and 70.38%, respectively.

###  Effect of Metformin and Dapagliflozin Treatment on TGF-β1, SMAD-3, PD-1, and NF- κB

**Figure 5:**
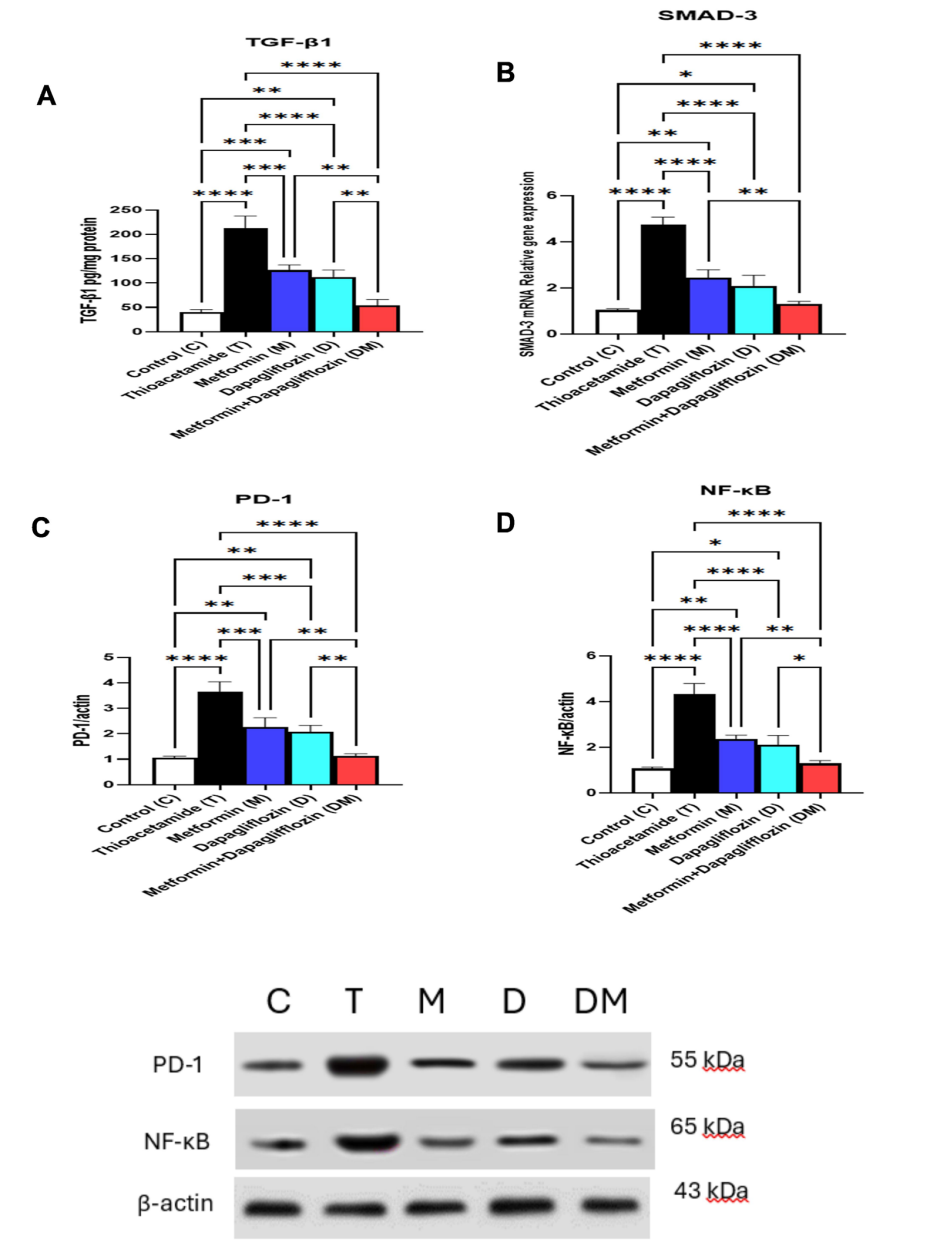
Effect of Metformin (M), Dapagliflozin (D), and their combination (DM) on the level of (A) TGF-β1, (B) SMAD-3 gene expression, (C) PD-1 protein level, and (D) NF-κB protein levels in rats with Thioacetamide-induced liver fibrosis. Data are presented as the mean ± SD (n = 6 per group). TGF-β1: Transforming growth factor β-1, SMAD-3: small mother against decapentaplegic-3, PD-1: Programmed death receptor, NF-κB: Nuclear factor kappa-light-chain-enhancer of activated B cells. ****P < 0.0001, ***P < 0.001, **P < 0.01, *P < 0.05. Control (C): normal rats received the CMC vehicle orally daily for one month; Thioacetamide (T): rats received thioacetamide (200 mg/kg) i.p. three times weekly for 4 weeks; Metformin (M): thioacetamide-induced liver fibrosis rats treated with oral metformin (300 mg/kg) daily for 4 weeks; Dapagliflozin (D): thioacetamide-induced liver fibrosis rats treated with oral dapagliflozin (1 mg/kg) daily for 4 weeks; The combination group (DM): thioacetamide-induced liver fibrosis rats treated with oral dapagliflozin (1 mg/kg) and oral metformin (300 mg/kg) daily for 4 weeks

Figure 5A shows that the TGF-β1 protein level was significantly increased in the liver tissue of rats of the thioacetamide group (5.29-fold) compared to the control group. At the same time, M, D, and DM reversed the thioacetamide effect (40.55%, 47.13%, and 74.37%), respectively. Besides, Figure 5B showed that the SMAD-3 relative gene expression was significantly increased in the thioacetamide group (4.57-fold) versus the control group, which was reversed by M, D, and DM by 48.48%, 56.18%, and 72.2%, respectively. Figure 5C shows that the PD-1 protein level was significantly increased in the liver tissue of rats of the thioacetamide group (3.44-fold) compared to the control group. At the same time, M, D, and DM reversed the thioacetamide effect (37.97%, 43.36%, and 69%), respectively. Besides, Figure 5D showed that the NF-κB protein level was significantly increased in the thioacetamide group (4-fold) versus the control group, which was reversed by M, D, and DM by 45.53%, 51.1%, and 70.15%, respectively.

###  Effect of Metformin and Dapagliflozin Treatment on Liver Functions 

**Figure 6:**
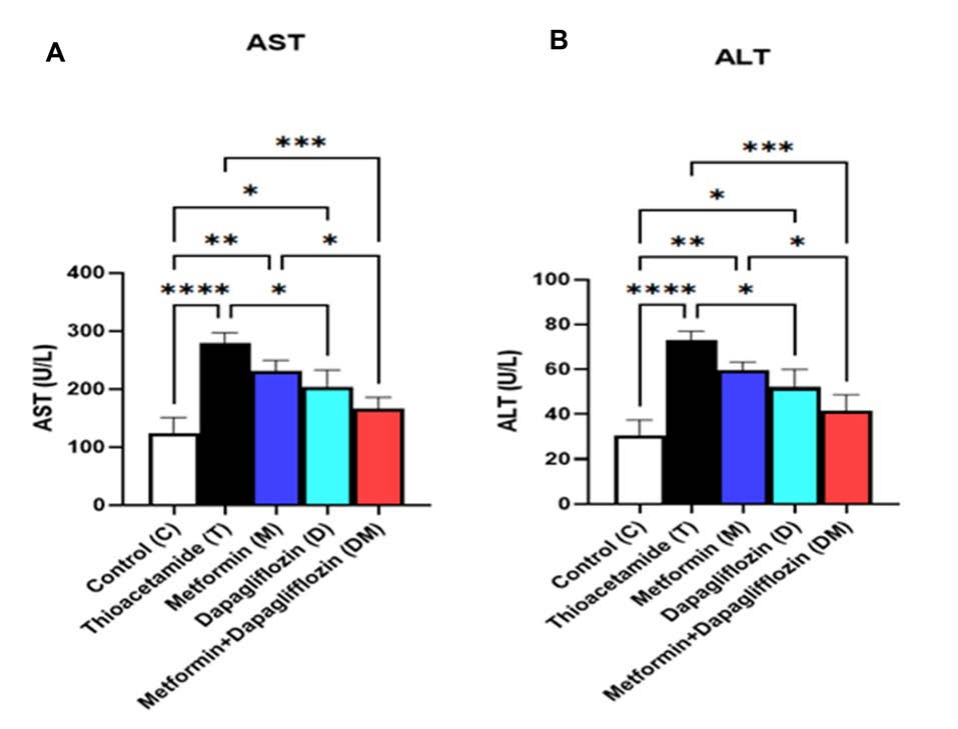
Effect of Metformin (M), Dapagliflozin (D), and their combination (DM) on the level of (A) AST and (B) ALT in rats with Thioacetamide-induced liver fibrosis. Data are presented as the mean ± SD (n = 6 per group). ALT: Alanine transaminase, AST: Aspartate transaminase. ****P < 0.0001, ***P < 0.001, **P < 0.01, *P < 0.05. Control (C): normal rats received the CMC vehicle orally daily for one month; Thioacetamide (T): Rats received thioacetamide (200 mg/kg) i.p. three times weekly for 4 weeks; Metformin (M): thioacetamide-induced liver fibrosis rats treated with oral metformin (300 mg/kg) daily for 4 weeks; Dapagliflozin (D): thioacetamide-induced liver fibrosis rats treated with oral dapagliflozin (1 mg/kg) daily for 4 weeks; The combination group (DM): thioacetamide-induced liver fibrosis rats treated with oral dapagliflozin (1 mg/kg) and oral metformin (300 mg/kg) daily for 4 weeks

Figure 6A shows that ALT activity was significantly increased in the liver tissue of rats of the thioacetamide group (2.4-fold) compared to the control group. At the same time, M, D, and DM reversed the thioacetamide effect (18.26%, 28.76%, and 42.9%), respectively. Besides, Figure 6B showed that AST activity was significantly increased in the thioacetamide group (2.25-fold) versus the control group, which was reversed by M, D, and DM by 17.4%, 27.17%, and 40.65%, respectively.

###  Effect of Metformin and Dapagliflozin Treatment on Oxidative Stress

**Figure 7:**
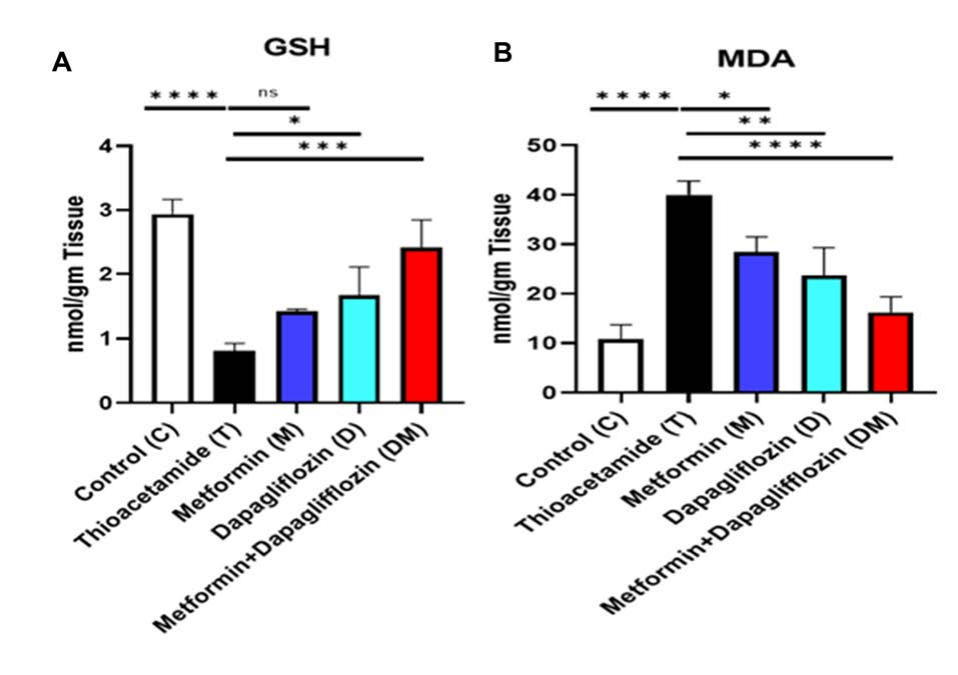
Effect of Metformin (M), Dapagliflozin (D), and their combination (DM) on the level of (A) MDA and (B) GSH in rats with Thioacetamide-induced liver fibrosis. Data are presented as the mean ± SD (n = 6 per group). MDA: Malondialdehyde, GSH: Reduced Glutathione. ****P < 0.0001, ***P < 0.001, **P < 0.01, *P < 0.05. Control (C): normal rats received the CMC vehicle orally daily for one month; Thioacetamide (T): Rats received thioacetamide (200 mg/kg) i.p. three times weekly for 4 weeks; Metformin (M): thioacetamide-induced liver fibrosis rats treated with oral metformin (300 mg/kg) daily for 4 weeks; Dapagliflozin (D): thioacetamide-induced liver fibrosis rats treated with oral dapagliflozin (1 mg/kg) daily for 4 weeks; The combination group (DM): thioacetamide-induced liver fibrosis rats treated with oral dapagliflozin (1 mg/kg) and oral metformin (300 mg/kg) daily for 4 weeks

Figure 7A shows that the MDA level was significantly increased in the liver tissue of rats of the thioacetamide group (3.69-fold) compared to the control group. At the same time, M, D, and DM reversed the thioacetamide effect (28.94%, 40.63%, and 59.39%), respectively. Besides, Figure 7B showed that the GSH protein level was significantly decreased in the thioacetamide group by 72.38% versus the control group, which was reversed by M, D, and DM by 1.75-fold, 2.06-fold, and 2.97-fold, respectively.

## Discussion

 The progression of chronic liver disease (CLD) toward liver cirrhosis and hepatic failure is driven by fibrogenesis and fibrosis, representing a maladaptive attempt to counteract persistent liver injury within the framework of the chronic wound healing response.^26^ Fibrotic remodeling stems from a fundamental imbalance between excessive extracellular matrix (ECM) deposition and insufficient ECM degradation, a dynamic that accelerates disease progression.^27^ Targeting myofibroblast differentiation, reducing senescent cell populations, and modulating the immune microenvironment represent a promising therapeutic strategy for reversing the pathological alterations associated with liver fibrosis.

 In this study, we explored the antifibrotic potential of dapagliflozin, metformin, and their combination in a TAA-induced liver fibrosis model in rats. Despite significant advancements in liver fibrosis research, key knowledge gaps persist, particularly regarding the intricate interplay between cellular senescence and immune checkpoint regulation and the potential synergistic antifibrotic effects of combined metformin and dapagliflozin therapy. In this study, we explore these questions by characterizing changes in senescence-related markers and features of immune microenvironment modulation within a well-established preclinical model of liver fibrosis.

 In this study, TAA-induced liver fibrosis was confirmed through histopathological examination, revealing portal fibrosis, congestion, and inflammation, with thick anastomosing fibrous tissue extensions. Treatment with metformin, dapagliflozin, or their combination effectively reversed these pathological alterations. Metformin-treated liver sections exhibited mild portal fibrosis and mild inflammation. Dapagliflozin-treated sections showed mild portal fibrosis, inflammation, edema, and congestion, indicating partial restoration of hepatic architecture. Combination therapy yielded the most profound effect, demonstrating fully repaired hepatic parenchyma with no signs of portal fibrosis.

 Additionally, Masson’s trichrome staining confirmed excessive collagen deposition in the TAA group and its significant reduction across all treatment groups. These histological findings were further supported by improvements in liver function markers and oxidative stress parameters, reinforcing the antifibrotic potential of these therapeutic interventions.

 In the present study, immunohistochemical analysis confirmed that fibroblast-to-myofibroblast transition was induced in rats following TAA administration, as evidenced by an increase in fibronectin and α-SMA, two key myofibroblast biomarkers.^28,29^ Importantly, treatment with metformin, dapagliflozin, or their combination significantly reduced fibronectin and α-SMA expression, supporting our histopathological findings.

 These results align with prior studies demonstrating the antifibrotic effects of metformin and dapagliflozin. Metformin decreases fibronectin and α-SMA expression in TGF-β1-induced nasal polyp-derived fibroblasts via gene and protein analysis.^30^ Similarly, dapagliflozin treatment significantly reduces α-SMA-positive cell populations through immunohistochemistry.^31^ These cumulative findings strongly reinforce our results regarding the dual-action antifibrotic effects of metformin and dapagliflozin in modulating fibroblast-to-myofibroblast transition.

 Transforming growth factor-β1 (TGF-β1) is a CLD key regulator, driving disease progression from initial liver injury to HCC.^32,33^ Elevated active TGF-β1 levels due to liver damage promote hepatocyte apoptosis and mediate hepatic fibroblast and hepatic stellate cell (HSC) activation, triggering a wound-healing response characterized by myofibroblast (MFB) formation and extracellular matrix (ECM) deposition. Given its central role as a profibrogenic cytokine, therapeutic targeting of the TGF-β pathway has been widely investigated as a strategy to halt liver disease progression.^34^ In addition, TGF-β1 has been implicated in the induction of cellular senescence across various cell types, including fibroblasts, bronchial epithelial cells, and certain cancer cells.^35,36^

 In the present study, TAA administration led to a significant upregulation of TGF-β1 protein levels, consistent with previous findings.^37,38^ However, treatment with metformin, dapagliflozin, or their combination effectively reversed this upregulation, supporting their antifibrotic potential. The modulation of TGF-β1 expression may explain the observed changes in myofibroblast differentiation, further corroborating reductions in fibronectin and α-SMA levels.^39-41^ Mechanistically, metformin has been shown to inhibit type II TGF-β1 receptor dimerization, thereby disrupting downstream fibrotic signaling.^42^ Furthermore, dapagliflozin significantly decreases TGF-β1 expression, as verified by gene and protein analysis, in a diabetes-induced diastolic dysfunction and cardiac fibrosis model.^43^ These findings reinforce the therapeutic potential of metformin and dapagliflozin in mitigating TGF-β1-mediated fibrogenesis, providing a mechanistic rationale for their antifibrotic effects

 The programmed death receptor (PD-1), which interacts with PD-L1, serves as a critical immunosuppressive regulator, playing a fundamental role in immune response modulation.^44^ In the current study, TAA administration led to upregulated PD-L1 protein levels, indicating a profound immunosuppressive effect. Notably, treatment with metformin, dapagliflozin, or their combination effectively reversed this increase, restoring immune function. These findings align with previous studies demonstrating the impact of TGF-β1 and PD-L1 modulation in carcinogenesis. TAA-induced intrahepatic cholangiocarcinoma (iCCA) in rats was associated with PD-L1 upregulation and increased CD8 + T cell infiltration, emphasizing the role of immune checkpoint disruption.^45^ AMP-activated protein kinase (AMPK) signaling plays a role in the mechanism by which metformin suppresses PD-L1 expression in tumor cells.^46^ Moreover, metformin-activated AMPK phosphorylates PD-L1 at S195, resulting in aberrant PD-L1 glycosylation and its subsequent accumulation in the endoplasmic reticulum (ER), triggering ER-associated degradation (ERAD).^47^

 Similarly, the FDA-approved sodium-glucose cotransporter-2 (SGLT2) inhibitor, canagliflozin, enhances T cell-mediated cytotoxicity while reducing PD-L1 expression, further supporting the immunomodulatory effects of metabolic modulators.^48^ The observed PD-L1 downregulation with metformin and dapagliflozin is likely linked to a concurrent reduction in cellular senescence, reinforcing their dual antifibrotic and immune-restorative properties.

 Elevated TGF-β1 levels contribute to NF-κB signaling activation, a pathway closely linked to inflammation and fibrosis, particularly in cardiac pathology.^49^ NF-κB is a transcription factor family comprising five members: NF-κB1 (p105/p50), NF-κB2 (p100/p52), p65 (RELA), RELB (V-Rel reticuloendotheliosis viral oncogene homolog B), and c-REL.^50^ Beyond fibrosis, NF-κB plays a critical role in SASP regulation, influencing immune recognition by natural killer (NK) cells, drug resistance, and cancer treatment outcomes. Global proteomic profiling has identified NF-κB as a master regulator of SASP gene expression during oncogene-induced senescence.^51^

 In the current study, TAA administration was associated with an accelerated senescence phenotype, evidenced by NF-κB upregulation. However, treatment with metformin, dapagliflozin, or their combination effectively reversed this increase, suggesting a critical role in senescence modulation. TAA triggers NF-κB signaling activation in liver tissue, reinforcing our findings.^52^ Metformin significantly reduces NF-κB gene and protein expression in mesangial cells (MCs) exposed to high glucose in a dose-dependent manner, highlighting its anti-inflammatory properties.^53^ Furthermore, dapagliflozin therapy lowers NF-κB p65 (pSer536) levels and reduces NF-κB p65 binding activity, further supporting its immunomodulatory and antifibrotic effects.^54^

 These findings underscore the therapeutic potential of metformin and dapagliflozin in mitigating NF-κB-driven inflammation and fibrosis, reinforcing their role in senescence and immune regulation.

 Cellular senescence is a permanent form of cell-cycle arrest caused by telomere shortening or cellular stress, and it is a key feature of aging tissues. The buildup of senescent cells is widely recognized as contributing to age-related disorders, with research showing that removing senescent cells can delay the development of age-associated diseases.

 Recent findings indicate a complex relationship between senescence and immune checkpoint regulation. Heterogeneous PD-L1 expression among senescent cells, with PD-L1⁺ senescent cells proliferating in vivo as they age.^55^ Despite the presence of SASP, PD-L1⁺ cells exhibit resistance to T-cell surveillance, whereas PD-L1⁻ cells remain susceptible. Single-cell in vivo analysis further linked elevated SASP levels with PD-L1 expression in p16⁺ cells. Notably, in naturally aging mice and in those with nonalcoholic steatohepatitis, PD-1 antibody treatment decreased both p16⁺ and PD-L1⁺ cell populations, mediated by activated CD8⁺ T cells, thereby improving various age-related phenotypes.

 Additionally, the Janus kinase (JAK)/signal transducer and activator of transcription (STAT) pathway is a critical mechanism through which senescent cells upregulate PD-L1 expression in non-senescent control cells.^56^ These insights reinforce the interplay between senescence, immune checkpoint regulation, and age-related pathologies, providing mechanistic links between immune dysfunction and fibrotic progression. In the current study, TAA administration was associated with accelerated senescence, as indicated by increased p16 expression and reduced levels of the antisenescence protein sirtuin 1 (SIRT1). Notably, treatment with metformin, dapagliflozin, or their combination effectively reversed these senescence-associated changes, suggesting their potential role in senescence modulation.

 Mechanistically, AMPK activation enhances mitochondrial biogenesis, activates PGC1-α, upregulates SIRT1, and suppresses NF-κB.^57^ Supporting these findings, metformin significantly reduces astrocyte senescence in Parkinson’s disease (PD) models, both in vivo and in vitro.^58^ Similarly, dapagliflozin decreases SGLT-2 expression and glucose consumption, preventing renal tubular epithelial cell senescence.^59^ These findings further support the role of SIRT1 in cellular senescence reversal, aligning with the observed antifibrotic effects of metformin and dapagliflozin.

## Conclusion

 Diabetes and aging are well-recognized risk factors for liver fibrosis, necessitating therapeutic interventions that simultaneously address metabolic dysfunction and fibrogenesis. Our findings highlight the multitarget antifibrotic potential of metformin and dapagliflozin, demonstrating their efficacy in mitigating biochemical and histopathological features of TAA-induced liver fibrosis.

 Treatment with metformin and dapagliflozin led to significant reductions in key profibrotic and immunosuppressive mediators, including TGF-β1, PD-1, p16, NF-κB, α-SMA, fibronectin, collagen, and oxidative stress biomarkers, while concomitantly elevating sirtuin-1 levels, reinforcing their senescence-modulating properties. These mechanistic effects translate into marked fibrosis regression, establishing metformin and dapagliflozin as promising antifibrotic agents, particularly for diabetic and elderly patients at heightened risk for progressive liver disease.

## Competing Interests

 The authors declare no conflict of interest.

## Data Availability

 The data that support the findings of this study are available from the corresponding author upon reasonable request.

## Ethical Approval

 All procedures were conducted in compliance with international ethical standards and approved by the Institutional Animal Care and Use Committee of Delta University for Science and Technology (Approval No. FPDu 15/2024), adhering to ARRIVE guidelines, EU Directive 2010/63/EU, U.K. Animals (Scientific Procedures) Act, 1986, and the NIH Guide for Care and Use of Laboratory Animals.
